# Integrated metagenomics and metabolomics reveal the dynamic mechanism in the rhizosphere soil of *Morus alba* L. and *Fraxinus mandshurica* Rupr. with *Inonotus hispidus*

**DOI:** 10.1128/aem.01251-25

**Published:** 2025-11-10

**Authors:** Qingchun Wang, Haiying Bao

**Affiliations:** 1 Key Laboratory for Development and Utilization of Fungi Traditional Chinese Medicine Resources, Jilin Agricultural University 85112, Changchun, China; 2 Key Laboratory of Edible Fungal Resources and Utilization (North), Jilin Agricultural University 85112, Changchun, China; University of Delaware, Lewes, Delaware, USA

**Keywords:** metagenomics, metabolomics, rhizosphere soil, *Morus alba* L., *Inonotus hispidus*, *Fraxinus mandshurica *Rupr.

## Abstract

*Inonotus hispidus* is a medicinal fungus that grows on *Morus alba* L., *Fraxinus mandshurica* Rupr., and *Ziziphus jujuba* Mill. However, there have been no reports on the comparative study of microbial diversity and metabolites in the rhizosphere soil of different tree species, specifically *M. alba* and *F. mandshurica*, growing with *I. hispidus*. Therefore, this study conducts a multi-omics joint analysis utilizing metagenomics and metabolomics to explore the differences in the synergistic mechanisms between different hosts of *I. hispidus*. Using metagenomics technology, a total of 177 phyla and 2,651 genera were identified as significantly different. At both the phylum and genus levels, Actinomycetota and Pseudomonadota as well as *Solirubrobacter* and *Bradyrhizobium* emerged as the predominant phyla and genera, respectively. In the Kyoto Encyclopedia of Genes and Genomes database, carbohydrate and amino acid metabolisms were identified as the primary metabolic pathways. Differential analysis of these metabolic pathways revealed that glucokinase and pyruvate kinase were downregulated. Additionally, metabolomics analysis identified 558 differential metabolites, with tyrosine metabolism being the foremost metabolic pathway involved. This pathway included five differential metabolites, among which salidroside, 3,4-dihydroxyphenylpropanoate, rosmarinate, and homovanillate were significantly upregulated in *M. alba* in association with *I. hispidus*. Furthermore, correlation analysis indicated that *Enhydrobacter* was positively correlated with 10 differential metabolites, while *Gaiella*, *Haladaptatus*, *Jiangella*, and *Prauserella* showed negative correlations. This study lays a solid foundation for elucidating the interactions between *I. hispidus* and its hosts, as well as for the effective utilization of *I. hispidus* resources across different tree species.

**IMPORTANCE:**

*Inonotus hispidus*, which is traditionally recognized as the authentic source of the medicinal fungus, primarily grows on *Morus alba* L. It is commonly found in ancient regions along the Yellow River, including Linqing, Xiajin, and Wudi in Shandong, as well as Chengde in Hebei Province and Aksu in Xinjiang. In traditional Chinese medicine, it is known as “Sanghuang” and has a long history of medicinal use. In addition to *M. alba*, *I. hispidus* also grows on other broad-leaved species, such as *Ulmus macrocarpa*, *Acer truncatum*, and *Fraxinus mandshurica*. The lack of fundamental research on its multi-host and -source diversity has hindered its industrial development and medicinal value. Consequently, this study employs metagenomics and metabolomics to investigate the rhizosphere soil microbial diversity and differential metabolites associated with the different host plants of *I. hispidus*, specifically *M. alba* and *F. mandshurica*, with the aim of providing a reference for its resource conservation and development.

## INTRODUCTION

*Inonotus hispidus*, a member of the family Hymenochaetaceae and the genus *Inonotus*, is a medicinal fungus documented in ancient Chinese medical texts, such as “Shennong’s Classic of Materia Medica” and “Compendium of Materia Medica.” Commonly referred to as “Sanghuang” in traditional Chinese medicine, its fruiting body predominantly grows on broad-leaved trees, including *Morus alba* L., *Ziziphus jujuba* Mill., *Ulmus macrocarpa* Hance., *Fraxinus mandshurica* Rupr., and *Malus pumila* Mill. This species is widely distributed across Northeast China, Northwest China, North China, East China, and Southwest China, with a long history of medicinal application (1, 2). Additionally, *I. hispidus* has been identified in South Korea, Japan, Canada, Germany, Mongolia, Algeria, and other regions. In 1996, hispolon, known for its potent antiviral and immunomodulatory properties, was first extracted from the fruiting body of *I. hispidus* (3). The fungus is also rich in various active compounds, including polysaccharides (4), polyphenols (5), terpenoids (6), flavonoids (7), and pigments (8). Modern research has demonstrated that *I. hispidus* exhibits a range of pharmacological activities, such as anti-tumor effects (9), alleviation of periodontitis (10), reduction of blood lipids (11), liver protection (12), immune enhancement (13), and inhibition of breast hyperplasia (14). While differences in the whole-genome sequence and secondary metabolites of *I. hispidus* from various tree species have been documented (15, 16), studies on the rhizosphere soil microbiome associated with different host plants of *I. hispidus* remain lacking. Therefore, this paper focuses on the rhizosphere soil of two host plants, *M. alba* and *F. mandshurica*, in association with *I. hispidus*, employing metagenomics and non-targeted metabolomics to elucidate the dynamic interactions between microorganisms and medicinal plants and uncover the molecular regulatory mechanisms underlying microbial diversity and secondary metabolites.

In recent years, the rapid advancement of high-throughput sequencing and metagenomics technologies has significantly propelled microbial research, offering an effective means to comprehend the complete diversity of environmental microbes. Among these studies, the rhizosphere soil microbial communities associated with certain edible fungi and their host plants have been analyzed, establishing a foundation for elucidating the interaction mechanisms between edible fungi and soil microorganisms. Consequently, the growth of edible fungi is closely linked to specific soil microbial communities, as evidenced by studies on species, such as *Agaricus bisporus*, *Morchella sextelata*, *Ganoderma leucocontextum*, *Stropharia rugosoannulata*, and *Phlebopus portentosus*. It is hypothesized that genera, such as *Sphingomonas*, *Mucilaginibacter*, *Bryobacter*, and *Bradyrhizobium*, may enhance the growth of *G. leucocontextum*. Notably, the relative abundance of these dominant beneficial bacteria diminishes with increasing cultivation years, which could be a significant contributing factor to the continuous cropping obstacles faced by *G. leucocontextum* (17). Additionally, *Acidobacterium* and *Acidisphaera* are recognized as key microorganisms involved in the formation of primordia during the cultivation of *Phlebopus portentosus*, while *Burkholderia* and *Bacillus* play a major role in the formation and maturation of fruiting bodies (18). Microorganisms involved in nitrogen fixation and nitrification, including *Arthrobacter*, *Bradyhizobium*, *Devosia*, *Pseudarthrobacter*, *Pseudolabrys*, and *Nitrospira*, are central to the symbiotic network in the cultivated soil of *M. sextelata* (19, 20). In the research concerning *I. hispidus*, previous studies have reported on the comparison of fungal and bacterial diversities among various host tree species (21). Additionally, metagenomic analyses of the rhizosphere soil microecology associated with different host plants of *I. hispidus* were conducted to further investigate the interactions between plants and rhizosphere microorganisms. This research aims to identify microecological regulation methods to enhance the quality of medicinal materials derived from *I. hispidus*.

With the advancement of the multi-omics technology, our understanding of the regulatory mechanisms of various bioactive molecules has significantly improved. Among these, metabolomics primarily focuses on the comprehensive analysis of metabolic small molecules and networks within organisms. Compared to transcriptome analysis, metabolomics allows for the rapid identification of differential metabolites across different samples, thereby enhancing our comprehension of biological phenomena. Literature indicates that the metabolic components of *I. hispidus* growing on five different tree species revealed a total of 1,353 metabolites with varying relative abundances (16). Researchers utilized ultra-performance liquid chromatography-quadrupole time-of-flight mass spectrometry (UPLC-Q-TOF-MS) to investigate the post-harvest dynamic processes of *A. bisporus* packaged with Nano-PM, identifying 35 differential metabolites (22). Furthermore, LC-MS/MS was employed to analyze the metabolomics of *M. sextelata* during storage, revealing that tyrosine metabolism plays a crucial role in the browning of *M. sextelata* over time (23). Additionally, hydrophilic interaction liquid chromatography with electrospray ionization and a quadrupole-time of flight mass spectrometer [HILIC-ESI (±)-Q-TOF-MS] and UPLC-Q-TOF-MS techniques were applied to explore the role of dopa melanin in the browning process of yellow cultivars of *Flammulina filiformis*, resulting in the identification of 107 metabolites that exhibited significant changes during browning (24). In light of this context, this study investigates the variation characteristics of differential metabolites and metabolic pathways in the rhizosphere soils of different host plants of *I. hispidus*, aiming to provide a scientific basis for understanding the regulation of the rhizosphere environment on metabolic networks and the response mechanisms of *I. hispidus* to the rhizosphere soils of various hosts.

This study employed metagenomics and metabolomics methods to investigate the microbial community structure and function in the rhizosphere soils of various host plants of *I. hispidus*. It also aimed to elucidate the molecular regulatory mechanisms of secondary metabolites. The primary objective was to gain a preliminary understanding of the interactions between *I. hispidus* and its host rhizosphere microorganisms. This research provides theoretical support for subsequent studies on the relationship between soil microorganisms and the formation of medicinal components in *I. hispidus* while also offering a novel perspective on the sustainable utilization of wild *I. hispidus* resources.

## MATERIALS AND METHODS

### Test materials and sample plot overview

**Fig 1:**
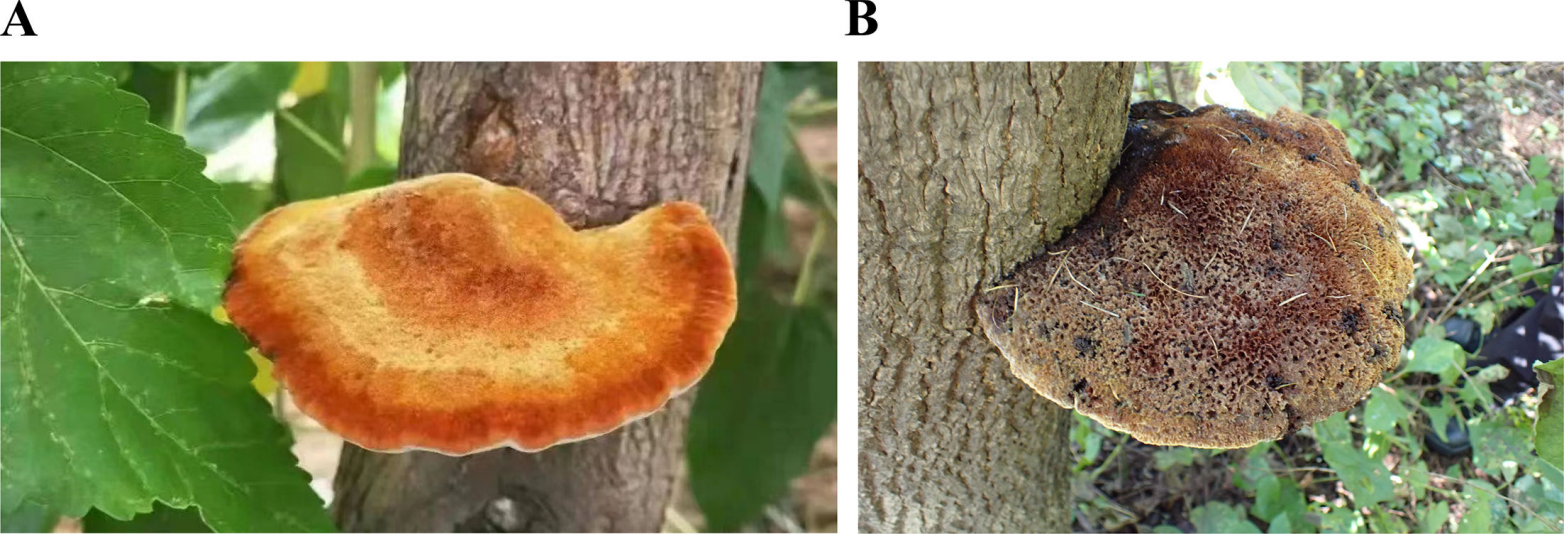
Habitat of *M. alba* and *F. mandshurica* growing *I. hispidus*. (**A**) *M. alba*. (**B**) *F. mandshurica*.

On 15 October 2023, we collected rhizosphere soil samples from *M. alba* in the ancient mulberry forest located in Xiajin County, Dezhou City, Shandong Province, China (36°59′ N, 115°11′ E) (Fig. 1A). The temperature on that day ranged from 11 to 23°C with no recorded rainfall. This area is situated in the warm temperate zone at an altitude of 23.5 to 34 m with an average annual temperature of 12.7°C and an average annual rainfall of 565.5 mm; the predominant soil type is brown soil. Additionally, we collected rhizosphere soil samples from *F. mandshurica* in Jingyuetan Forest Park, Changchun City, Jilin Province, China (43°79′ N, 125°46′ E) on 12 October 2023 (Fig. 1B). The temperature in this region ranged from 6 to 12°C, also with no rainfall. It is located in the middle temperate zone with an altitude of 220 to 406.5 m, an average annual temperature of 4.8°C, and an average annual rainfall of 654.3 mm; the soil here is classified as black soil. Soil samples from the 0 to 20 cm layer were collected using a five-point sampling method in an “S” shape. After mixing the soil samples, the quartering method was employed to remove impurities from the retained samples using a mesh sieve with a diameter of 2 mm. The samples were then placed into 50 mL sterile centrifuge tubes, preserved in dry ice for transport back to the laboratory, and stored in a refrigerator at −80°C for future analysis. The metagenomic samples of the rhizosphere soil from *M. alba* and *F. mandshurica* were designated as MARSM and FMRSM, respectively, while the metabolomics samples were labeled as MARSU and FMRSU. Each sample was replicated six times.

### Metagenomic analysis of rhizosphere soil

The initial step involved extracting genomic DNA from environmental samples, followed by assessing its integrity through 1% agarose gel electrophoresis. The DNA was subsequently fragmented using a Covaris M220, targeting an approximate size of 400 bp. A paired-end (PE) library was then prepared utilizing the NEXTflex Rapid DNA-Seq Kit (Bioo Scientific, USA). Sequencing and bridge PCR were performed using NovaSeq or Hi Seq X reagent kits (Illumina, USA). Gene prediction of open reading frames (ORFs) within the assembled contigs was conducted using prokaryotic gene prediction tools, Prodigal (25) and MetaGene (26). Genes containing nucleic acid sequences larger than 100 bp were compiled and translated into proteins. To construct a non-redundant gene set, CD-HIT (27) was employed to analyze the predicted gene sequences across all samples, selecting the longest sequence within each cluster. Cleaned RNA-seq reads from each sample were aligned to the non-redundant gene set using SOAPaligner (28), and gene abundance in each sample was evaluated. The non-redundant gene set was further compared against the NR database using BLASTP v22.28+ with an *e*-value cut-off of 1e−5. Species annotations were derived from the taxonomic table associated with the NR database, and species quantities were determined by summing the genes relevant to each species. To create tables for each of the four taxonomic ranks—phylum, family, genus, and species—abundance data for taxa were collected (29). The DIAMOND tool (30) was utilized for BLASTP analysis against the Carbohydrate-Active Enzymes (CAZy) database. Additionally, using the BLASTP option, the non-redundant gene sequences were compared with the Kyoto Encyclopedia of Genes and Genomes (KEGG) database, with the *e*-value set to 1e−5. For functional annotation of the predicted genes, KOBAS 2.0 software (KEGG Orthology-based Annotation System) was employed, incorporating these alignment details.

### Metabolomics analysis of rhizosphere soil

The soil samples were filtered and subsequently freeze-dried under vacuum for 48 h. The dried samples were then ground into a powder using a freeze grinding machine at a frequency of 30 Hz for 1.5 min. A quantity of 100 mg of the powdered sample was weighed and dissolved in 0.6 mL of 70% methanol extract. The mixture was vortexed six times and centrifuged at 10,000 × *g* for 10 min. The supernatant was collected and further filtered through a 0.22 µm microporous membrane before being placed into a clean injection vial, which was stored at 4°C in a refrigerator for the UPLC-MS/MS experiment. Chromatographic analysis was conducted using an Acquity UPLC HSS T3 column (1.8 µm, 2.1 × 100 mm). The mobile phase consisted of phases A (0.1% formic acid in water) and B (acetonitrile). The proportion of phase B was increased from 2 to 50% over the first 10 min, raised to 95% by 11 min, and maintained for 2 min before returning to 2% and holding for an additional 15 min. The flow rate was set at 400 µL/min with an injection volume of 2 µL. UPLC separation was followed by analysis with a SCIEX 6500 QTRAP triple quadrupole mass spectrometer equipped with an IonDrive Turbo V ESI ion source. The mass spectrometer operated in multiple reaction monitoring (MRM) mode with an ion spray voltage of 5,500 V and a source temperature of 400°C. The pressures in both ion source gas I (GSI) and II (GSII) were maintained at 60 psi. The mass spectrometry data were further analyzed using the SCIEX Analyst Work Station Software (Version 1.6.3). The preprocessed data were annotated using the KEGG compound database (https://www.kegg.jp/kegg/compound). Additionally, the *T* value fold changes (FC) of individual metabolites were calculated. OPLS-DA was performed in SIMCA (V14.1) with 200 permutations to ensure model quality. Subsequently, variable importance in projection (VIP) values were calculated, and metabolites were considered significantly different if the *P*-value was ≤0.05 and VIP > 1. Therefore, metabolites with log2(FC) ≥ 1 were classified as upregulated, whereas those with log2(FC) ≤ −1 were classified as downregulated. The KEGG pathway database (https://www.kegg.jp/kegg/pathway.html) was utilized to analyze the functional pathways of these differential metabolites (31).

### Correlation analysis of metagenomics and metabolic data

This study compared the levels of differential metabolites and identified key differential genera in the rhizosphere soil of both *M. alba* and *F. mandshurica* to assess how these relative differences influenced their correlation. The Pearson correlation coefficients between the relative abundance of each key differential genus and eight significant metabolites at the genus level were calculated using the Pearson method. A Sankey diagram was created with the ggalluvial package in R. Additionally, microbial co-occurrence network analysis was performed using Gephi 9.2 (https://gephi.org). The Mantel test was conducted utilizing the mantel function available in the vegan package of R.

## RESULTS

### Composition of microbial community

**Fig 2:**
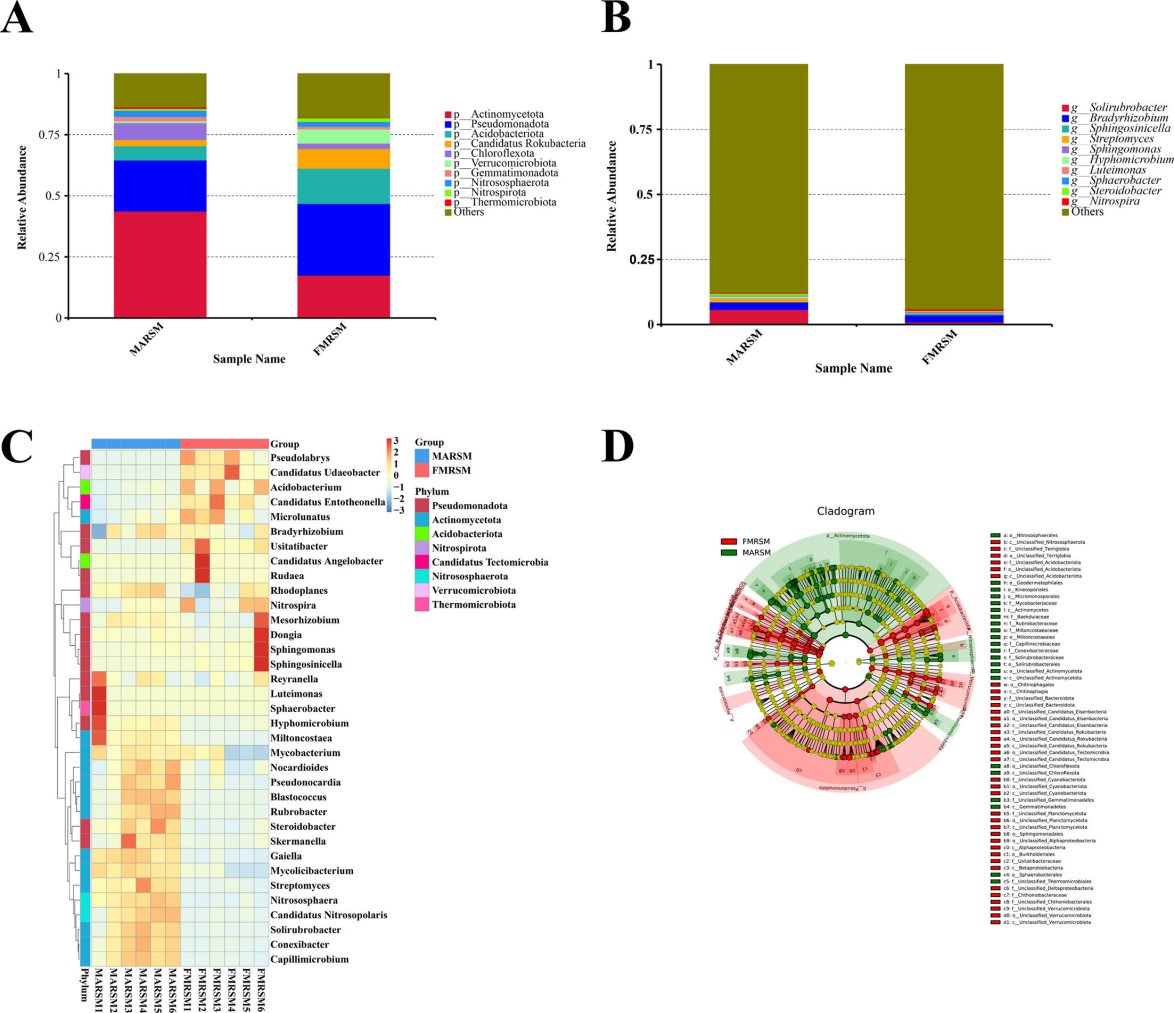
Species annotation. (**A**) Phylum-level relative abundance histogram. (**B**) Genus-level relative abundance histogram. (**C**) Genus-level relative abundance clustering heat map. (**D**) LDA value evolutionary branch map.

In comparison to other fungi, including but not limited to *Thelephora ganbajun* (32), *Leucocalocybe mongolica* (33), *Agaricus sinodeliciosus* (32), and *Inocybe terrigena* (34), it was observed that, although the dominant groups and their proportions differed from the results, the overall community composition remained largely consistent. This similarity may be attributed to the varying sequencing techniques employed. Furthermore, the study utilized metatranscriptome methods to sequence the mycorrhizal tissues of *Piloderma* fungi and pine trees, integrating genomic data analysis to identify the most highly expressed coding transporter protein of *Piloderma* (35). We conducted phylum and genus annotations on two rhizosphere soil samples, MARSM and FMRSM. The findings indicated that Actinomycetota and Pseudomonadota were the two predominant taxa among the top 10 species annotations at the phylum level. The relative abundance of Actinomycetota in MARSM and FMRSM was 71.58 and 28.42%, respectively, while that of Pseudomonadota in MARSM and FMRSM was 41.52 and 58.13%, respectively. Consequently, the distribution proportion of Actinomycetota in MARSM was greater than that in FMRSM, whereas Pseudomonadota was enriched in FMRSM and less prevalent in MARSM (Fig. 2A).

At the genus level, a total of 2,651 genera were annotated, with *Solirubrobacter* and *Bradyrhizobium* showing significant enrichment. A relatively higher quantity of MARSM was observed compared to FMRSM. In the outcome analysis of MARSM and FMRSM, many of the ORFs were categorized as unclassified (others) (Fig. 2B and C). *Solirubrobacter* belonging to the Actinobacteria phylum is widely distributed in soil, particularly in dry and semi-arid regions. It exhibits strong resistance to drought and salinity, allowing it to survive under extreme conditions. Additionally, *Bradyrhizobium*, a class of nitrogen-fixing bacteria within the Alphaproteobacteria, has the ability to oxidize complex organic compounds. The results indicated that the rhizosphere soil of *M. alba* and *F. mandshurica* possessed higher organic matter content and nitrogen fixation capability, enabling *I. hispidus* to access a more abundant nutrient supply in a soil environment characterized by a complex microbial community structure.

The LEfSe results indicated that there were 68 and 73 potential characteristic microbial classifications in MARSM and FMRSM, respectively. The specific differences are primarily evident in the greater activity of unclassified Actinomycetota at the class and order levels, as well as the species Actinomycetota bacterium, unclassified Chloroflexota at both the class and order levels, and various other species in MARSM. In contrast, FMRSM exhibited enrichment of the genus Candidatus Entotheonella, the species Pseudolabrys taiwanensis, the genus Candidatus Angelobacter, the species Candidatus Angelobacter sp-Gp1-AA117, and the species Chthoniobacterales bacterium, among other species (Fig. 2D).

### CAZymes and KEGG function database annotation of MARSM and FMRSM

**Fig 3:**
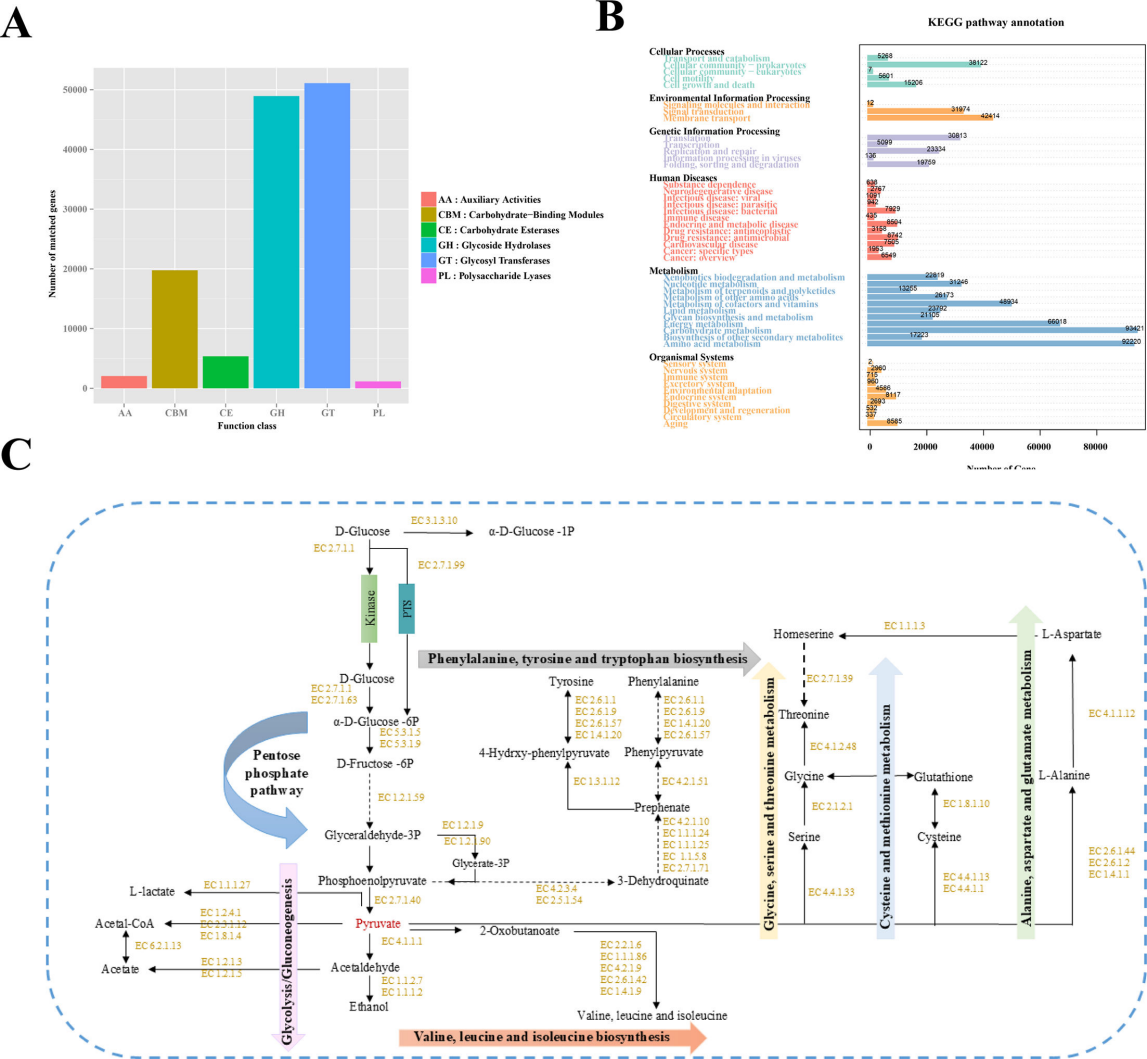
Functional database annotations of MARSM and FMRSM. (**A**) Functional database annotations of the CAZy database. (**B**) Functional database annotations of the KEGG database. (**C**) Effects of MARSM and FMRSM on carbohydrate and amino acid metabolism pathway.

CAZymes significantly influence the formation and maturation of edible fungi by decomposing organic matter, providing carbon sources, and regulating morphogenesis and immunity. The growth and development of edible fungi involve the continuous accumulation of nutrients. Through CAZymes, cellulose, hemicellulose, lignin, and other substances are converted into carbon sources essential for growth. Based on the CAZymes database, a total of 128,368 genes were annotated in FMRSM and MARSM (Fig. 3A). These genes are classified into glycosyl transferases (GT), glycoside hydrolases (GH), carbohydrate esterases (CE), auxiliary activities (AA), polysaccharide lyases (PL), and carbohydrate-binding modules (CBM). These enzymes can assemble or degrade carbohydrates (such as starch and cellulose), which are widely present in nature. Among them, GTs and GHs represent the two most significant types of CAZyme genes comprising 39.80 and 38.13% of the total gene count, respectively. Notably, GT8 and GH13 exhibited significant differences (*P* < 0.05). GT8 is a type of GT that functions as a lipopolysaccharide α-1,3-galactose transferase (EC: 2.4.1.44) with polysaccharides as its substrate, while the GH13 family is responsible for degrading complex carbohydrates. This supports the conclusion that GHs and GTs are the most abundant CAZymes. This suggests that the microorganisms of *I. hispidus* may play an essential role in the formation of rhizosphere soils of different host plants through the degradation of polysaccharides and insoluble dietary fibers mediated by GHs and GTs. It is noteworthy that both samples contained families GH2, GH5, GH8, GH13, GH30, and GH99, which are associated with pectin, cellulose, hemicellulose, and starch. This indicates that the growth of *I. hispidus* fruiting bodies is closely linked to the degradation of EC: 3.2.1.21 (β-glucosidase), EC: 3.2.1.23 (β-galactosidase), EC: 3.2.1.78 (β-1,4-mannanase), EC: 3.2.1.4 (cellulase), EC: 3.2.1.1 (α-amylase), and α-1,2-mannoside.

The KEGG database was utilized to annotate six level 1 metabolic pathways. Among these pathways, metabolic biological processes were the most prominent, accounting for 65.20% of the total genes. The analysis also identified five other pathways, that is, genetic information processing, environmental information processing, cellular processes, human diseases, and organismal systems, which contributed 10.50, 9.87, 8.52, 6.66, and 3.91% of the total genes, respectively. In total, 46 metabolic pathways were assigned at level 2 (Fig. 3B). Based on the KEGG annotation, the metabolic differences between MARSM and FMRSM were examined. The significantly enriched metabolic pathways in both groups were similar, yet the abundance of enzymes within these pathways varied significantly. Notably, EC: 3.2.1.41 (branched-chain amylase) exhibited significantly different abundances across the ko00500 pathway (starch and sucrose metabolism) (*P* < 0.05), with MARSM showing higher abundance compared to FMRSM. This enzyme facilitates the conversion of UDP-glucose to α-D-Glu-1-P. The ko00500 pathway is crucial for glycogen degradation, converting glycogen into glucose-6 phosphate, which is subsequently broken down into acetyl-CoA and pyruvate. Acetyl-CoA enters the TCA cycle via the ko00020 pathway, producing oxaloacetic acid and 2-oxoglutarate. Additionally, EC: 4.2.1.9 (dihydroxyacid dehydratase) displayed significantly different abundances in the ko00029 pathway (biosynthesis of valine, leucine, and isoleucine) (*P* < 0.05), with MARSM again showing higher levels compared to FMRSM. This enzyme catalyzes the conversion of (R)-2,3-dihydroxy-3-methylbutyrate into valine, leucine, and isoleucine, linking it to the mutual transformation within the ko00260 pathway (glycine, serine, and threonine metabolism).

### Effects of MARSM and FMRSM on carbohydrate metabolism and amino acid metabolism

Based on the metagenomic data, further investigations were conducted on the metabolic pathways. Research has revealed that the most frequently annotated genes are those associated with carbohydrate and amino acid metabolism. Carbohydrate metabolism, which encompasses the glycolytic pathway, carbohydrate transport, and the citric acid cycle, plays a crucial role in energy production and cellular biosynthesis in anaerobic systems. The carbohydrate-related metabolic processes were analyzed through a three-level annotation system, and network analyses were performed on the glycolysis and amino acid biosynthesis pathways (Fig. 3C). Glycolysis is regarded as the primary pathway for carbohydrate metabolism linked to glucose conversion and energy generation. Notably, genes encoding enzymes for carbohydrate utilization are predominantly enriched in lactic acid bacteria. Within the glycolytic metabolic pathway, α-D-glucose-1P and D-glucose are converted to α-D-glucose-6P by the enzymes EC: 2.7.1.1 and EC 2.7.1.63. Subsequently, under the influence of EC: 5.3.1.5 and EC 5.3.1.9, α-D-glucose-6P is further degraded to D-fructose-6P, which subsequently generates β-D-fructose-1,6P2. Following the production of α-D-glucose-6P and α-D-fructose-6P, these compounds are further utilized through the subsequent glycolysis and pyruvate metabolic pathways to produce acids and substrates for amino acid metabolism. Therefore, MARSM and FMRSM may utilize glucose for glycolysis, leading to the formation of pyruvate, which serves as a vital precursor for the production of organic acids, ethanol, and various amino acids.

Recently, Olmedo et al*.* (36) employed proteomics and metabolomics to investigate the pathways associated with carbon metabolism in *Vitis vinifera* fruit and its center following treatment with cytokinin [N-(2-chloro-4-pyridyl)-N′-phenylurea (CPPU)]. Their findings revealed an increase in the abundance of enzymes involved in the Embden-Meyerhof-Parnas (EMP) pathway and the tricarboxylic acid (TCA) cycle in grapefruit treated with CPPU. Additionally, variations in related substances and carbohydrates were observed across different metabolic pathways. Graham et al. (37) identified that the central carbon metabolic pathway is intricately linked to carbohydrate decomposition, with malate synthase (MS) and isocitrate lyase (ICL) serving as key enzymes in the glyoxylate and acetic acid (GAC) cycle. These enzymes facilitate intracellular metabolic reactions and act as specific developmental signals that regulate the GAC cycle, thereby influencing lipid storage during the early growth stage of plant seed post-germination. Pyruvate metabolism serves as a fundamental basis for substrate conversion within metabolic pathways. In the glucose to pyruvate pathway, the relative abundance of EC: 2.7.1.2 (glucokinase) and EC: 2.7.1.40 (pyruvate kinase) significantly decreases. This reduction in the relative abundance of glucokinase and pyruvate kinase results in a decreased level of pyruvate, which subsequently inhibits lactic acid production, thereby lowering soil pH and creating a more favorable environment for the growth of *I. hispidus*. This phenomenon also contributes to the reduced acid production observed in the two strains of *I. hispidus*. Phosphoenolpyruvate is degraded to pyruvate by EC: 2.7.1.40 and EC: 2.7.9.2 (pyruvate, water dikinase) and further converted to acetyl-CoA by EC: 1.2.4.1, EC: 2.3.1.12, and EC: 1.8.1.4 (dihydroacyl dehydrogenase). Additionally, acetic or pyruvic acid can be converted into acetaldehyde, which can then be transformed into ethanol, representing another more abundant metabolite.

In the context of amino acid metabolism, pyruvate serves as a crucial precursor for amino acid biosynthesis. Pyruvic acid directly generates alanine, serine, leucine, isoleucine, valine, and cysteine, which subsequently lead to the formation of other amino acids, such as tyrosine, phenylalanine, lysine, proline, and arginine. Notably, proline and arginine can be synthesized via the urea cycle. Pyruvate facilitates the production of leucine, valine, and isoleucine through the action of EC: 2.2.1.6 (acetolactate synthase) and the enzymes EC: 1.1.1.86, EC: 4.2.1.9, EC: 2.6.1.42, and EC: 1.4.1.9 (branched-chain aminotransferase). These amino acids are ultimately degraded into fatty acid-derived aldehydes and alcohols, such as nonanol, pentanol, and octanol. Consequently, this paper posits that *I. hispidus* can utilize these amino acids present in rhizosphere soil as growth substrates to enhance its symbiotic relationships and facilitate nitrogen fixation. Among these amino acids, phenolic acids are derived from the phenylalanine metabolic pathway, which contributes to the abundance of phenolic acids in *I. hispidus*. Additionally, prephenate is converted into phenylalanine and tyrosine through the actions of EC: 2.6.1.9 (phosphohistidine aminotransferase) and EC: 1.3.1.12 (prephenate dehydrogenase).

Consequently, the central carbon metabolic pathway, also referred to as energy metabolism, consists of the EMP pathway, TCA cycle, and PPP, with pyruvate serving as the primary connecting substrate. It is interconnected with protein, lipid, nucleic acid, organic acid, and secondary metabolite metabolisms, influencing the overall life processes of plants. This underscores the potential for future research on the central carbon metabolism pathway in plant rhizosphere soil to leverage the comprehensive integration of multiple omics technologies to accurately elucidate the regulatory networks governing the central carbon metabolism pathway. Such an approach would reveal the interactions and dynamic changes among metabolites, enzymes, and genes within the central carbon metabolism pathway across different plant species, growth stages, and stress environments. In summary, metagenomic sequencing technology was employed to analyze the functional dynamics of microbial communities in both MARSM and FMRSM. The results indicated that MARSM significantly enhanced the activity of carbohydrate-active enzymes and the metabolic processes of key metabolic pathways in rhizosphere soil. This enhancement facilitated carbon metabolism and promoted denitrification, nitrate reduction, and glutamate synthesis within the nitrogen cycle of rhizosphere soil. These physiological activities are closely associated with the degradation of proteins and cellulose, as well as the fixation of carbon dioxide. Overall, MARSM and FMRSM exhibit an upregulatory effect on aminopeptidase and transaminase, which may influence the biosynthesis of amino acids in *I. hispidus*. However, it is important to note that while this technology can identify genes necessary for specific functions within the microbial community’s genome, the mere presence of these genes does not confirm their expression. Therefore, further investigations utilizing macrotranscriptome and macroproteome analyses are warranted.

### Change characteristics of the rhizosphere soil metabolite spectrum

**Fig 4:**
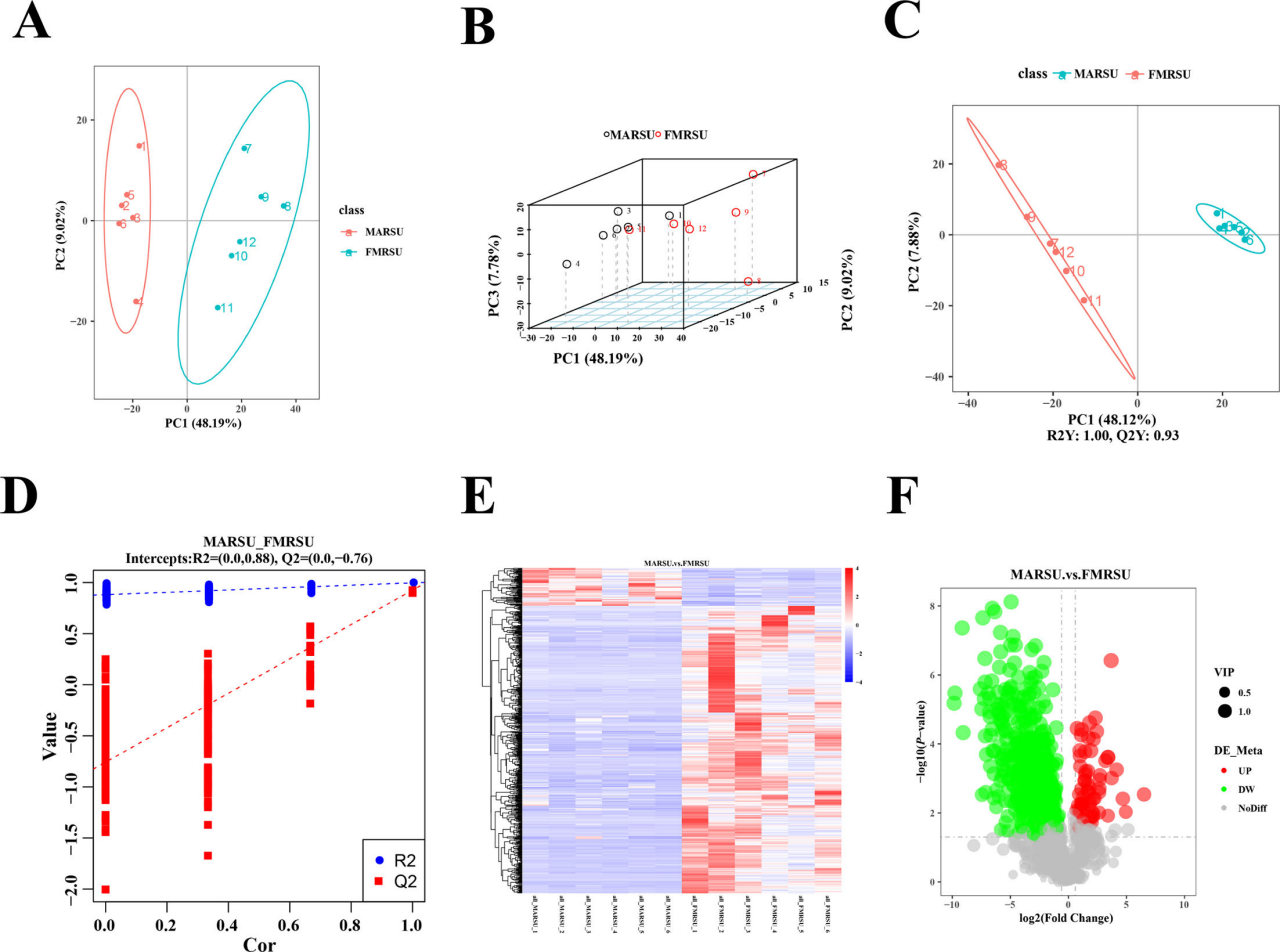
MARSU and FMRSU PCA analysis, PLS-DA, and screening of differential metabolites. (**A, B**) PCA analysis. (**C, D**) PLS-DA analysis of the 200-permutation test model validation. (**E**) Heat map. (**F**) Volcano diagram of differential metabolites.

To assess the reliability of the instrument, this study employed the UPLC-MS/MS method. A quality control sample (QC) created by mixing the sample extracts was utilized as the analytical sample to evaluate the repeatability of the detection system under consistent treatment conditions. The total ion current (TIC) of the QC samples was overlaid to generate an ion current map for the multi-substance extraction of metabolites. The results indicated that the retention time and peak intensity of the mass spectrum peaks for the QC sample were consistent, with a high degree of curve overlap, suggesting that the instrument exhibits good signal stability for the same sample across different detection times. Two distinct clusters emerged from the rhizosphere soil groups of MARSU and FMRSU, with all quality control samples clustering together, thereby demonstrating good analytical stability and repeatability. Notably, MARSU displayed a more concentrated and similar distribution along the PC1 and PC2 dimensions. In contrast, FMRSU was distinctly separated from MARSU in the PC1 (48.19%) dimension, although both host plants formed separate clusters in the PC2 (9.02%) dimension (Fig. 4A and B). Given the high dimensionality and strong correlations among variables in the metabolome data, traditional univariate analysis is insufficient for rapidly and accurately extracting potential information from the data. Consequently, we applied partial least squares discriminant analysis (PLS-DA) to further investigate the differences in metabolite composition within the rhizosphere soil of the different tree species, MARSU and FMRSU. The results were consistent with those obtained from the principal component analysis (PCA). The scatter plot from the PLS-DA score principal component analysis revealed R2Y = 1.00 and Q2Y = 0.93, indicating the formation of two distinct clusters between the MARSU and FMRSU groups, which suggests that the PLS-DA model effectively captures the differences in metabolite composition between these two groups (Fig. 4C). The permutation test yielded Q2 = −0.76 < 0 and *R*2 = 0.88, indicating that the PLS-DA model is not overfitted (Fig. 4D).

### Screening of differential metabolites and enrichment analysis of metabolic pathways

A total of 831 metabolites were successfully annotated and confirmed in the MARSU and FMRSU samples. The analysis employed one-way analysis of variance (ANOVA) and utilized the Benjamini-Hochberg method to identify differentially expressed metabolites with a significance threshold of *P* < 0.05. Subsequently, metabolic visualization was conducted, resulting in a clustered heat map that illustrated the abundance relationships among the metabolites (Fig. 4E). A horizontal comparison was employed to elucidate the relationships between the abundance clusters of the metabolites across the two samples, revealing a relative distribution of metabolite levels. Additionally, the volcano plot of the differential metabolites indicated variations in metabolite concentrations between MARSU and FMRSU (Fig. 4F).

**Fig 5:**
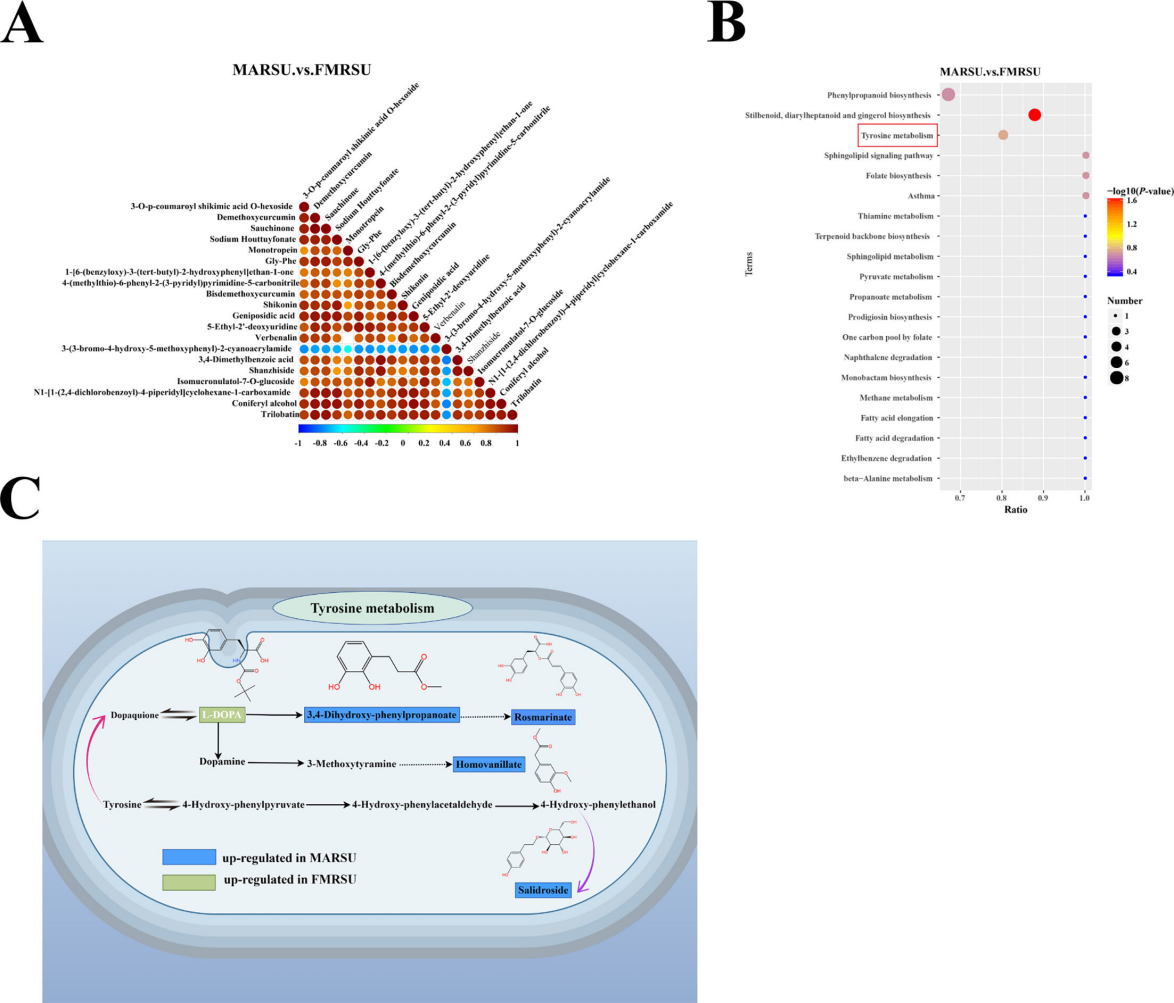
MARSU and FMRSU metabolite annotation results. (**A**) Differential metabolite correlation diagram. (**B**) KEGG enrichment bubble diagram. (**C**) Tyrosine metabolic pathway.

To analyze the synergistic or mutually exclusive relationships among different metabolites, we calculated the Pearson correlation coefficient for the top 20 differential metabolites out of 558 based on ascending *P*-values (*P* < 0.05) (Table S1). For instance, 3-O-p-coumaroyl shikimic acid O-hexoside exhibited a positive correlation with 19 compounds, including demethoxycurcumin, sauchinone, sodium houttuyfonate, monotropein, and Gly-Phe, while showing a negative correlation with 3-(3-bromo-4-hydroxy-5-methoxyphenyl)-2-cyanoacrylamide (Fig. 5A). Furthermore, we enriched pathways to explore the biological functions of these metabolites, identifying the fundamental biochemical metabolic and signal transduction pathways associated with the differential metabolites. Following KEGG pathway enrichment, we identified 73 pathways, including stilbenoid, diarylheptanoid, and gingerol biosynthesis, tyrosine metabolism, folate biosynthesis, phenylpropanoid biosynthesis, ethylbenzene degradation, terpenoid backbone biosynthesis, fatty acid metabolism, and steroid hormone biosynthesis (Fig. 5B). Notably, tyrosine metabolism, the most significantly enriched pathway, involved five differential metabolites, among which salidroside, 3,4-dihydroxyphenylpropanoate, rosmarinate, and homovanillate were significantly upregulated in MARSU, while 3,4-dihydroxy-L-phenylalanine was significantly upregulated in FMRSU (Fig. 5C). According to the literature, salidroside is a natural phenolic compound that has been shown to exert positive regulatory effects on inflammation, oxidative stress, apoptosis, and tissue damage during cancer, cardiovascular, liver, and kidney diseases. Thus, it is speculated that MARSU is rich in phenolic acids. Conversely, 3,4-dihydroxy-L-phenylalanine, also known as levodopa, is significantly upregulated in FMRSU and serves as a precursor for the synthesis of norepinephrine and dopamine in the body. Due to the complexity of its metabolite sources, its biochemical metabolic and signal transduction pathways are intricate and diverse, encompassing the host’s own metabolites, microbial metabolites, and co-metabolites from both host and microbes. Nevertheless, this complexity provides a material basis for the adaptation of *I. hispidus* to various host ecological environments and the nutrient and energy cycles in the rhizosphere. The resultant differential accumulation may influence the optimal conditions for the growth of the *I. hispidus* fruiting body.

### Relationship between rhizosphere soil microbial diversity and key differential metabolites

**Fig 6:**
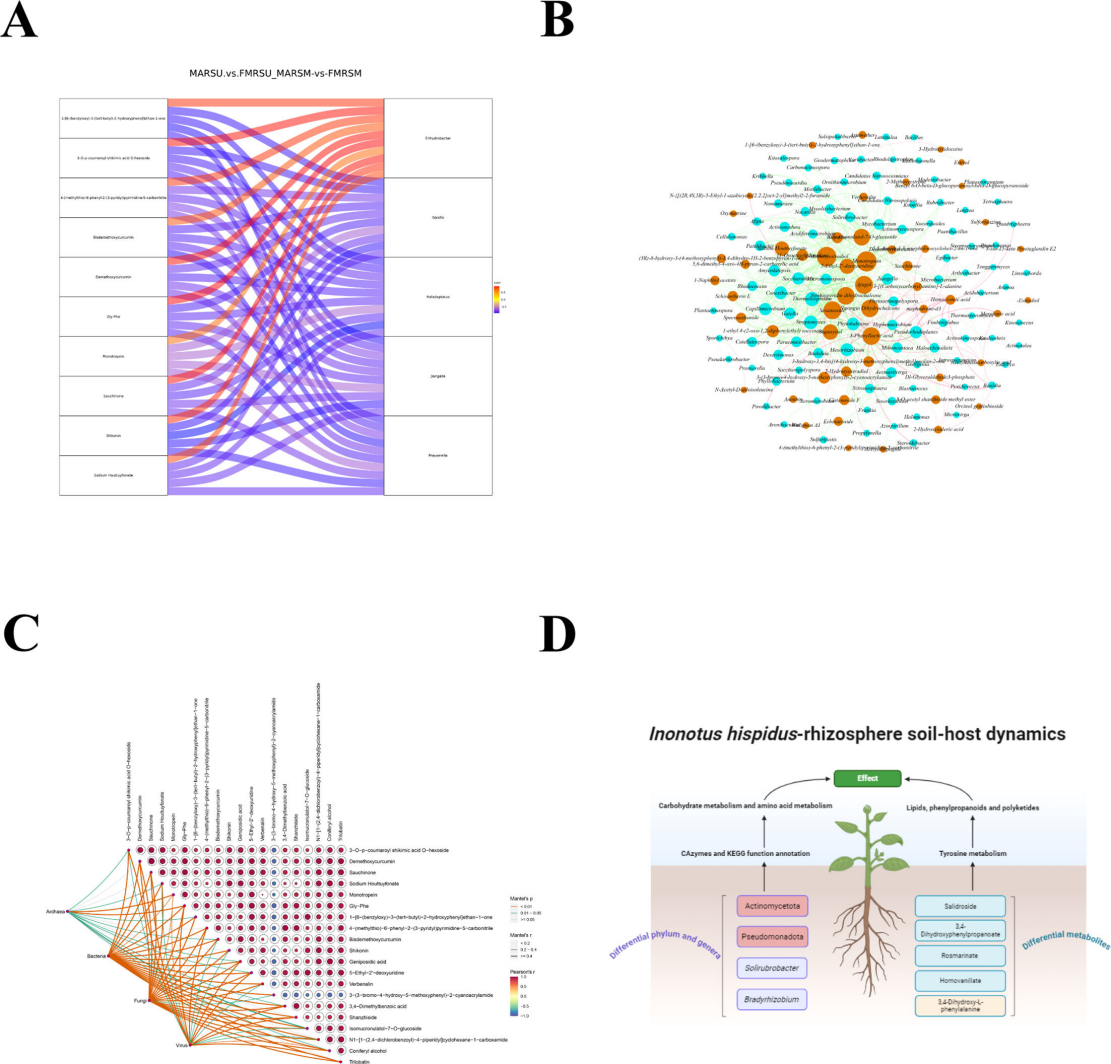
Association analysis of metagenomics and metabolomics. (**A**) Sankey diagram. (**B**) Correlation network analysis of differential metabolites and rhizosphere microbial communities. (**C**) Mantel test analysis. (**D**) Mechanism of *I. hispidus*-rhizosphere soil-host dynamics.

The interaction among plants, soil, and microorganisms is crucial for maintaining ecosystem balance. Due to the distinct genetic backgrounds and metabolic processes of various plants, the secretions in their rhizospheres differ, leading to diverse rhizosphere microenvironments. An increasing body of evidence indicates that the structure and function of the rhizosphere soil microbiome significantly influence plant development. We analyzed the correlation between two groups of the top five microorganisms: *Enhydrobacter*, *Gaiella*, *Haladaptatus*, *Jiangella*, and *Prauserella*. The results revealed that *Enhydrobacter* was positively correlated with several compounds, including 1-[6-(benzyloxy)-3-(tert-butyl)-2-hydroxyphenyl]ethan-1-one, 3-O-p-coumaroyl shikimic acid O-hexoside, 4-(methylthio)-6-phenyl-2-(3-pyridyl)pyrimidine-5-carbonitrile, bisdemethoxycurcumin, demethoxycurcumin, Gly-Phe, monotropein, sauchinone, shikonin, and sodium houttuyfonate. In contrast, *Gaiella*, *Haladaptatus*, *Jiangella*, and *Prauserella* exhibited negative correlations. Based on these findings, a Sankey diagram was created (Fig. 6A). An analysis of the topological indices from the collinearity network of bacterial and fungal communities showed that the number of network edges and the average degree of the *M. alba* rhizosphere soil were higher than those of *F. mandshurica* rhizosphere soil, with both exhibiting elevated modularity. This indicates that the rhizosphere soils of *M. alba* and *F. mandshurica* enhance the correlation between bacterial and fungal communities, resulting in a more modular and stable fungal community structure (Fig. 6B). A Mantel test was conducted to evaluate the correlation between differential metabolites and microbial communities. The results demonstrated that the top 20 differential metabolites, including 3-(3-bromo-4-hydroxy-5-methoxyphenyl)-2-cyanoacrylamide, monotropein, sodium houttuyfonate, verbenalin, 3-O-p-coumaroyl shikimic acid O-hexoside, shanzhiside, and demethoxycurcumin, significantly affected the fungal community (*P* < 0.05) (Fig. 6C). A multi-omics-driven gene regulatory network was constructed for a deeper exploration of the dynamic mechanisms underlying the interactions among *I. hispidus*, rhizosphere soil, and host dynamics (Fig. 6D).

The effective substances involved in the rhizosphere soil metabolism are crucial for enhancing interactions between microorganisms and plants. Additionally, microbial communities serve as significant driving factors for soil metabolism. Our study reveals that the relationship between rhizosphere soil metabolites and bacterial communities is more pronounced than that with fungal communities. We propose that the influence of rhizosphere soil metabolites on fungal community structure is greater than on bacterial communities, with bacteria playing a more pivotal role in modifying rhizosphere metabolites. Joint analysis indicates a strong correlation between microorganisms with differential abundance and metabolites with differential abundance, suggesting that microorganisms may interact with metabolites or that metabolites may influence the relative abundance of microorganisms for stress adaptation. The top 10 metabolites exhibiting the greatest differences include phenols and fatty acids, which align with the secondary metabolites of *I. hispidus* reported previously (16). Our findings demonstrate that these metabolites are significantly enriched alongside differentially abundant microorganisms within the KEGG pathway. This suggests that phenolic substances influence the microbial composition involved in the formation of fruiting bodies in crude filamentous fungi. Notably, several diverse microbial genera, such as *Gaiella*, *Haladaptatus*, *Jiangella*, and *Prauserella*, are detrimental to plant growth. This implies that the low abundance of *Enhydrobacter* may benefit the growth of these microorganisms. Although we have speculated about the relationship between certain metabolites and microorganisms, the interactions among microorganisms remain to be thoroughly investigated.

In conclusion, significant differences were observed in the microbial community structure and metabolites in the rhizosphere soil of *M. alba* and *F. mandshurica* associated with different tree species hosting *I. hispidus*. These differences are not solely attributed to variations in biological factors but are also influenced by practical considerations, such as sampling techniques, weather conditions, sample preservation, and data sequencing depth. All of these factors impact the information obtained regarding microbial community structure and metabolomics analysis. In future research, genomics, proteomics, transcriptomics, and metabolomics, along with other gene regulatory networks, will be employed to construct a comprehensive gene regulatory network. This approach will facilitate the interpretation of the growth and development processes, such as sampling techniques, weather conditions, sample preservation, and data sequencing depth. All of these factors impact the information obtained regarding microbial community structure and metabolomics analysis. In future research, genomics, proteomics, transcriptomics, and metabolomics, along with other gene regulatory networks, will be employed to construct a comprehensive gene regulatory network. This approach will facilitate the interpretation of the growth and development processes of *I. hispidus* at the genomic, proteomic, transcriptional, and metabolic levels. Furthermore, it will enable a detailed exploration of the regulatory mechanisms and causal relationships among various molecules through the integration of multi-omics data, thereby elucidating the interaction mechanisms between fungi, rhizosphere soil, and their host. These methodologies will aid in identifying and targeting regulatory genes, providing valuable insights into the generation of fungal signals and the specific responses of rhizosphere biota.

### Conclusion

Metagenomic and metabolomic analyses were employed to characterize the microorganisms and metabolites present in the rhizosphere soil where *I. hispidus* predominantly grows in *M. alba* and *F. mandshurica*. LEfSe analysis of the bacterial and fungal taxa revealed significant genera, including *Solirubrobacter* and *Bradyrhizobium*. In terms of metabolic pathways, the two samples exhibited the most significant annotations in carbohydrate and amino acid metabolisms, along with a notable upregulation of the GT8 gene. Furthermore, the mechanisms underlying these metabolic pathways demonstrated an upregulation of related enzymes involved in glycolysis and pyruvate and amino acid metabolisms. *Enhydrobacter* was found to be positively correlated with 10 differential metabolites. Additionally, metabolomics identified a total of 562 differential metabolites, which were associated with multiple metabolic pathways, primarily focusing on the accumulation of lipids and lipid-like molecules, phenylpropanoids, polyketides, and organic oxygen compounds. Notably, tyrosine metabolism was significantly enriched, with salidroside and 3,4-dihydroxy-L-phenylalanine being significantly upregulated in MARSU and FMRSU, respectively. This paper proposes a future multi-omics-driven construction of gene regulatory networks to further explore the dynamics between *I. hispidus*, rhizosphere soil, and their host, thereby providing a foundation for the development and utilization of wild resources of *I. hispidus* from various sources.

## Data Availability

The authors confirm that the data supporting the findings of this study are available within the article and its supplemental material. The metagenomics data of rhizosphere soil of *Morus alba* L. and *Fraxinus mandshurica* Rupr. with *Inonotus hispidus* have been deposited in the NCBI Sequence Read Archive (SRA) under accession no. PRJNA1280255 and PRJNA1280262, respectively, and will become publicly available once accession formalization resumes.

## SUPPLEMENTAL MATERIAL

Table S1 aem.01251-25-s0001.xlsx Differential metabolites of MARSU and FMRSU.

ASM does not own the copyrights to Supplemental Material that may be linked to, or accessed through, an article. The authors have granted ASM a non-exclusive, world-wide license to publish the Supplemental Material files. Please contact the corresponding author directly for reuse.
